# Influence of iron and copper consumption on weight gain and oxidative stress in adipose tissue of Wistar rats

**DOI:** 10.2478/v10102-012-0021-6

**Published:** 2012-09

**Authors:** Alexey A. Tinkov, Olga P. Ajsuvakova, Alexandr M. Shehtman, Viktor M. Boev, Alexandr A. Nikonorov

**Affiliations:** 1 Department of Biochemistry, Orenburg State Medical Academy, Orenburg, Russia; 2 Interdepartmental Biochemical Laboratory, Orenburg State Medical Academy, Orenburg, Russia; 3 Department of Human Pathology, 1st Orenburg Regional Clinical Hospital, Orenburg, Russia; 4 Department of general and communal hygiene and human ecology, Orenburg State Medical Academy, Orenburg, Russia

**Keywords:** metals, oxidative stress, weight gain, rat

## Abstract

The aim of the present study was to assess the effect of iron and copper consumption on weight gain and development of oxidative stress in adipose tissue of rats. Control rats obtained pure drinking water. Iron-treated groups of animals obtained FeSO_4_•12H_2_O with drinking water in concentrations of 3 and 6 mg/l, while copper-treated rats obtained CuSO_4_ in concentrations of 4.88 and 9.76 mg/l. The animals of the 6th group received a mixture of FeSO_4_•12H_2_O and CuSO_4_ in the respective concentrations of 3 and 4.88 mg/l in drinking water. All animals received a standard chow. The final weight of rats from all the experimental groups, especially in those obtaining the combination of iron and cooper, exceeded the control values. Maximal weight of fat pads was observed in animals receiving drinking water with 3 mg/l FeSO_4_•12H_2_O, 4.88 and 9.76 mg/l CuSO_4_, and the mixture of FeSO_4_•12H_2_O and CuSO_4_. The maximal intensity of free radical processes, as estimated by the concentration of fluorescent modified amino acids and the intensity of chemiluminescence in adipose tissue homogenates, was observed in rats obtaining iron in the concentration of 3 mg/l in the drinking water.

## Introduction

Obesity has been recognized as a global epidemic by the end of the 20th century (James 2008). Officially it is defined as an excess of body adiposity (Caballero, 2007). According to classic theories, obesity develops due to high-caloric intake, physical inactivity and genetic predisposition (Bailie-Hamilton, 2002). In the past decade, the role of ecology in obesity induction has been investigated. Numerous widely spread chemicals, predominantly polyaromatic hydrocarbons and polychlorinated biphenyls, are able to cumulate in adipose tissue (Mullerova & Kopecky, 2007) and induce weight gain (Irigaray *et al.*, 2006; Arsenescu *et al.*, 2008) in cases of impaired neural or humoral regulation (Bailie-Hamilton, 2002). At the same time it is known that obesity is characterized not only by growing mass of adipose tissue but also by development of oxidative stress and inflammation (Suzuki *et al.* 2003; Nadal-Casellas *et al.*, 2011). Moreover, it is supposed that activation of free radical oxidation processes is one of the mechanisms leading to impaired adipose tissue signaling and subsequent increase of fat depots (Evans *et al.*, 2003; Grimsrud *et al.*, 2007). Oxidative stress itself can be caused by redox-metals, which are widely spread in the environment. Though there are data indicating iron-induced impairment of adipose tissue signaling (Dongiovanni *et al.*, 2011), the ability of d-metals to induce weight gain has only partially been studied (Tajima *et al.*, 2011; Tinkov *et al.*, unpublished results). The aim of our research was to assess the effect of iron and copper consumption on weight gain and development of oxidative stress in adipose tissue of rats.

## Materials and methods

### Chemicals

Bovine serum albumin was obtained from Sigma (St. Louis, USA), Sodium di-hydrogen Phosphate 2-hydrate and di-Sodium Hydrogen Phosphate 2-hydrate for phosphate buffer were obtained from Panreac (Barcelona, Spain). All other chemicals used were of analytical or higher purity.

### Animals

The current research was performed according to the rules of the Local Ethics Committee. One-month-old female Wistar rats (n=36) were divided into six groups. Female rats were used due to the physiologically larger content of adipose tissue than present in males. This anatomical feature provides the opportunity to obtain more representative results. The light and the dark cycles in the animal room were 12 hours each. The rats had been acclimatized to the laboratory conditions for one week before the study began. All animals were fed ad libitum. A granulated chow (“Orenburg food mixture factory”, Orenburg, Russia) containing 270 kcal/100g (20% protein, 70% carbohydrate, 10% fat) was used as the diet. Food consumption was registered daily at the same time. Body weight was determined once a week. After 90 days of experiment, the rats were sacrificed by decapitation.

### Treatment

All groups of animals, except the control group, received FeSO_4_·12H_2_O and CuSO_4_ with drinking water. The concentrations of iron and copper used were calculated on the basis of maximum permissible concentrations (MPC) for these chemicals in the Russian Federation and were consequently ecologically relevant. MPCs for single chemicals Fe^2+^ and Cu^2+^ are 0.3 and 1.0 mg per liter of drinking water, respectively. Rats of the first group were used as controls and received high-quality drinking water with general mineralization less than 250 mg/l (“Aqua Vita”, Orenburg, Russia; certified by the A. N. Sysin Research Institute of Human Ecology and Environmental Health). Animals of the 2^nd^ and the 3^rd^ groups received drinking water containing 3 and 6 mg/l FeSO_4_·12H_2_O, respectively. Rats of the 4^th^ and the 5^th^ groups consumed CuSO_4_ with water in concentrations of 4.88 and 9.76 mg/l, respectively. Animals from the 6^th^ group received the mixture of FeSO_4_·12H_2_O and CuSO_4_ in the respective concentrations of 3 and 4.88 mg/l in drinking water. With the purpose of creating natural conditions, the animals were given an unlimited access to water. Water consumption was measured daily with the use of graduated bottles. The daily measurement showed no significant difference in water consumption of animals of the control and of the experimental groups.

### Morphometric studies

Body length and the final weight were determined before dissection and were used to calculate the body mass index (BMI) according to the equation (Novelli *et al.*, 2007):

BMI = body weight (g) / length^2^ (cm^2^)

Hair of the rats was cut from the dorsal surface for subsequent analysis of copper and iron concentration in hair. During dissection, parametrial and periovarial fat was removed. The total weight of these fat pads was measured immediately. Harvested parametrial adipose tissue was used for histologic evaluation, determination of iron and copper content, and of oxidative stress markers, oxidatively modified amino acids, and chemiluminescence in particular.

### Histological evaluation

Fragments of harvested parametrium were fixed in 10% neutral formalin solution, embedded in paraffin and then stained with hematoxylin and eosin. In the obtained histological sections cellular content of adipose tissue was evaluated visually.

### Iron and copper assay

Hair analysis was used for determination of metal content since the concentration of the elements in hair reflects the influence of environmental factors and correlates with the concentration of chemical elements in other tissues of the organism (Creason *et al.*, 1975). The dissected parametrium was used also for determination of metals in adipose tissue. Iron and copper in harvested tissues were determined by the respective methods of atomic-emission and mass-spectrometry with inductively coupled plasma (µg/g of tissue). For elemental content of tissues, determination spectrometer Optima 2000 DV (Perkin-Elmer, USA) and spectrometer Elan 9000 (Perkin-Elmer, USA) were used.

### Fluorescence studies

Oxidatively modified amino acids in adipose tissue were determined fluorometrically in order to assess the intensity of protein oxidation. Adipose tissue was homogenized in 20 volumes of ice cold phosphate buffer. The final volume of probes for analysis was 2 ml and contained 1.9 ml of 1/15 phosphate buffer (pH=7.4) and 0.1 ml of the adipose tissue homogenate. All probes were diluted to the final protein concentration of 5µg/ml. The protein concentration was determined by the method of Lowry (Lowry *et al.*, 1951) using bovine serum albumin as standard. The formation of dityrosines was evaluated by measuring the emission spectra (380–440 nm) at excitation wavelength 325 nm (slit width 5 nm) (Giulivi & Davies, 1994). Fluorescence emission spectra were measured in the range of 420–480 nm in order to determine the concentration of conjugates of lipid peroxidation products with free amino groups of proteins, primarily lysine (Lys-LPO) (Dousset *et al.*, 1994). The excitation wavelength was 365 nm (slit width 5 nm). The concentration of oxidatively modified amino acids was expressed in relative fluorescent units (RFU). All fluorescence measurements were performed on Varian Cary Eclipse spectrofluorometer (Varian Inc., Australia) at 25 °C.

### Chemiluminescence studies

The intensity of chemiluminescence was measured on Chemiluminomer-003 (Ufa, Russia) in order to assess the level of free radical oxidation processes in adipose tissue. Adipose tissue was homogenized in 20 volumes of isopropanol. An aliquot of 0.25 ml was added into the tube containing 4 ml of heptane-isopropanol solution (1:1). The probe (final volume 4.25 ml) was incubated for 20 minutes and after addition of 0.5 ml distilled water centrifuged at 3 000×g for 10 minutes. The upper heptane phase was pipetted for subsequent analysis since it is assumed that heptane extracts neutral lipids, which present the major lipid fraction of the adipocyte lipid drop. The final volume in the cuvette was 5 ml containing 4.9 ml of heptane-isopropanol mixture and 0.1 ml of the upper phase. After stabilization of the signal, the inductor FeSO_4_·7H_2_O was entered into the system. After induction of free-radical processes in the system, fast flash amplitude and general luminosity were recorded. Fast flash amplitude characterizes the intensity of reactive oxygen species generation as a response to Fe^2+^ addition and indirectly shows the concentration of hydroperoxides in serum. General luminosity serves as an indicator of free radical oxidation of biomolecules in serum. The intensity of chemiluminescence was expressed in conventional units (c.u.) of luminosity (Lopukhin *et al.*, 1983).

### Statistical analysis

The data were expressed as mean values ± SEM and evaluated using Mann-Whitney U-test at the significance level 2 alpha = 0.05.

## Results

### Weight gain and food consumption

Table 1 shows that the mass of all animals increased during the experiment. The intensity of weight gain during the first month was similar in the control and the experimental groups. By the end of the second month, the mean weight values of rats became relatively higher in all experimental groups compared to controls. The maximal weight gain was registered in animals obtaining the mixture of FeSO_4_·12H_2_O and CuSO_4_ in the respective concentrations of 3 mg/l and 4.88 mg/l. At the end of the experiment, the maximal weight gain was observed in rats obtaining 6 mg/l FeSO_4_·12H_2_O, 4.88 mg/l CuSO_4_, and the combination of FeSO_4_·12H_2_O and CuSO_4_ in the above mentioned concentrations.

**Table 1:** Intensity of food consumption and weight gain in rats.

No	Obtained chemical (mg/l)	Initial weight (g)	Food consumption during 1st month (g)	Weight after 1st month (g)	Food consumption during 2nd month (g)	Weight after 2nd month (g)	Food consumption during 3d month (g)	Weight after 3d month (g)	Total weight gain (g)
1	Control (–)	173.0±1.8	18.63±0.81	237.8±1.8	18.33±0.43	284.4±1.9	19.15±0.55	291.6±1.7	118.6±2.3
2	Fe^2+^ (3)	172.5±4.5	19.85±1.40	236.7±4.9	18.89±0.40	285.9±6.5	19.33±0.78	292.0±8.1	119.5±4.6
3	Fe^2+^ (6)	176.8±1.9	18.50±1.21	239.1±4.0	18.91±0.62	294.0±7.0	19.54±1.46	302.6±6.0	125.8±6.7
4	Cu+ (4.88)	169.8±2.2	22.15±1.38	232.5±4.4	22.31±0.78*	287.5±5.8	22.81±1.39	297.0±5.6	127.2±6.1
5	Cu^2+^ (9.76)	174.2±4.2	20.22±0.99	243.8±2.1	19.54±0.51	285.8±3.0	19.24±0.91	298.7±3.0	124.5±4.3
6	Fe^2+/^Cu^2+^ (3/4.88)	171.8±3.2	20.78±1.11	244.9±1.8	20.43±0.67	296.3±4.8	21.29±0.73	308.7±4.7*	136.9±5.7*

All data represented as Mean ± SEM

* p < 0.05 *vs* control group of animals

Food consumption dynamics increased in animals receiving metals with drinking water during the experiment. The maximal food consumption was observed in rats obtaining water containing 4.88 mg/l CuSO_4_ and the combination of FeSO_4_·12H_2_O and CuSO_4_ in the respective concentrations of 3 and 4.88 mg/l.

### Morphometric parameters of rats

Table 2 shows that the mean values of fat pad weight were higher in the experimental groups of animals than in controls. The maximal weight of fat pads was observed in animals receiving drinking water with 3 mg/l FeSO_4_·12H_2_O, 4.88 and 9.76 mg/l CuSO_4_, and the mixture of FeSO_4_·12H_2_O and CuSO_4_ in the above mentioned concentrations. In these groups the mean values of this parameter exceeded control values by 45–60%. BMI was relatively higher in the experimental groups than in controls but the difference was not significant.

**Table 2:** Rat morphometric parameters.

N^2^	Obtained chemical (mg/l)	Fat pad weight (g)	Percentage of fat pad weight from body weight (%)	BMI
1	Control (–)	4.97±0.13	1.70±0.04	0.65±0.01
2	Fe^2+^ (3)	7.50±0.51*	2.62±0.12 *	0.69±0.02
3	Fe^2+^ (6)	6.17±0.37	2.03±0.15	0.70±0.02
4	Cu+ (4.88)	7.43±0.27*	2.50±0.13 *	0.69±0.01
5	Cu^2+^ (9.76)	7.22±0.42 *	2.41±0.12 *	0.69±0.01
6	Fe^2+/^Cu^2+^ (3/4.88)	7.95±0.67 *	2.57±0.21 *	0.67±0.01

All data represented as Mean ± SEM

* *p<*0.05 *vs* control group of animals

### Iron and copper content in tissues

The obtained data in Table 3 show that the concentration of iron and copper in tissues depends on the incoming dose. The maximal concentration of iron in rat hair was observed in animals obtaining 6 mg/l iron with drinking water, exceeding control values by 20%. Iron content in groups of animals receiving 3 mg/l FeSO_4_·12H_2_O and the mixture of FeSO_4_·12H_2_O and CuSO_4_ in the above mentioned concentrations also exceeded the control group values, though this difference was not significant. Concentration of copper in hair was significantly higher in experimental animals receiving drinking water containing 9.76 mg/l of CuSO_4_ than in controls. It is important to mention that an increase of copper content in rat hair was observed in all experimental animals compared to controls, especially in animals receiving the metal mixture, although this increase was not significant.

**Table 3:** Iron and copper content in hair and adipose tissue of rats.

No	Obtained chemical (mg/l)	Concentration of metals in hair (µg/g)		Concentration of metals in adipose tissue (µg/g)	

		Fe	Cu	Fe	Cu
1	Control (–)	19.31±0.67	12.32±0.37	6.62±0.81	0.27±0.02
2	Fe^2+^ (3)	21.83±0.63	12.93±0.40	7.63±0.70	0.23±0.03
3	Fe^2+^ (6)	22.93±1.21*	13.11±0.54	10.24±1.45*	0.25±0.02
4	Cu+ (4.88)	19.79±0.31	13.54±1.01	8.28±2.29	0.27±0.06
5	Cu^2+^ (9.76)	18.67±2.11	13.77±0.27*	8.75±0.97	0.24±0.02
6	Fe^2+/^Cu^2+^ (3/4.88)	21.14±2.40	14.25±1.14	7.06±0.36	0.27±0.02

All data represented as Mean ± SEM

* *p<*0.05 *vs* control group of animals

As seen in Table 3, the most notable difference in the concentration of metals in adipose tissue was observed in the case of iron. In hair of animals receiving 6 mg/l FeSO_4_·12H_2_O with drinking water, the iron content in adipose tissue was significantly higher by 55% than in controls.. It is important to notice that in animals receiving 4.88 and 9.76 mg/l CuSO_4_, the concentration of iron in adipose tissue was higher than in controls by 25 and 32%, respectively, though the difference was not significant. In case of copper, no significant increase in adipose tissue was observed.

### Oxidative stress markers in adipose tissue

As seen in Table 4, fluorescence of dityrosines in animals receiving drinking water containing iron in either concentration and in the combination of FeSO_4_·12H_2_O and CuSO_4_ exceeded control values by approximately 25%. Fluorescence of Lys-LPO was higher than in controls in all experimental groups, while the maximal fluorescence exceeded control values by 50% in animals obtaining 3 mg/l FeSO_4_·12H_2_O and CuSO_4_ in both concentrations.

**Table 4:** Markers of oxidative stress in adipose tissue homogenates.

N^2^	Obtained chemical (mg/l)	Dityrosine, RFU	Lys-LPO, RFU	Fast flash amplitude (c. u.)	General luminosity (c. u.)
1	Control (–)	0.79±0.06	0.59±0.04	0.66±0.03	1.66±0.05
2	Fe^2+^ (3)	1.00±0.05*	0.95±0.09*	1.75±0.27*	2.17±0.12*
3	Fe^2+^ (6)	0.97±0.09	0.67±0.12	0.73±0.03	1.83±0.09
4	Cu+ (4.88)	0.79±0.07	0.87±0.09*	0.71±0.05	1.80±0.14
5	Cu^2+^ (9.76)	0.79±0.10	0.88±0.05*	0.73±0.07	2.07±0.11*
6	Fe^2+/^Cu^2+^ (3/4.88)	0.94±0.05*	0.71±0.05*	0.87±0.15	2.07±0.13*

All data represented as Mean ± SEM

* *p<*0.05 *vs* control group of animals

Mean values of chemiluminescent parameters were enhanced in groups of animals receiving metals. The maximal intensity of flash amplitude was observed in animals with drinking water containing the combination of FeSO_4_·12H_2_O and CuSO_4_ and FeSO_4_·12H_2_O in the concentration of 3 mg/l. In the last case a 3-fold increase in the fast flash amplitude was recorded. Mean values of general luminosity were also higher in the experimental groups than in controls. The maximal general luminosity was measured in adipose tissue of rats obtaining 3 mg/l of FeSO_4_·12H_2_O, 9.76 mg/l of CuSO_4_, and the combination of iron and copper. The data exceeded control values by 30, 25 and 25%, respectively.

### Adipose tissue morphology

**Figure 1:**
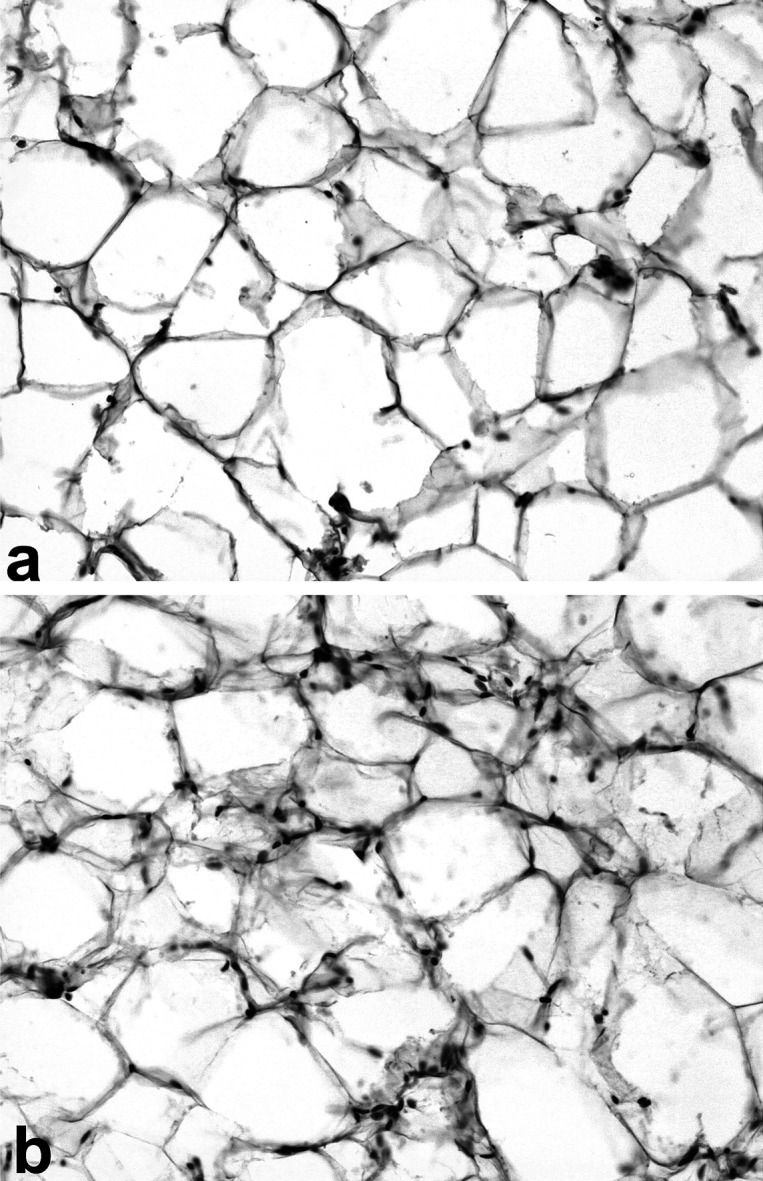
Histology of rat adipose tissue. Hematoxylin and eosin stains (×200). (a) adipose tissue of control rat. Only single lymphoid cells are present; (b) adipose tissue of rats obtaining FeSO_4_·12H_2_O and CuSO_4_ in respective concentrations of 3 and 4.88 mg/l. Lymphoid infiltration of adipose tissue is observed.

The histological evaluation of adipose tissue revealed lymphoid infiltration of parametrium in all experimental groups. It is important to mention that the intensity of lymphoid infiltration revealed practically no difference between research groups. The most representative control and experimental slices of adipose tissue are shown in Figure 1.

## Discussion

The obtained data substantiate the following conclusions: i) consumption of iron and copper with drinking water led to excessive weight gain and increase of adipose tissue mass in comparison with control rats; ii) consumption of iron and copper with drinking water resulted in elevated levels of metals in rat hair; iii) entrance of metals into the organism led to iron accumulation in adipose tissue, whereas copper content in adipose tissue did not change; iv) on entering the organism, the above mentioned metals induced oxidative stress and inflammation in adipose tissue.

All the observed effects of metal consumption are undoubtedly the consequence of their cumulation in the organism. In case of single metal consumption, the concentrations of iron and copper in hair seem to depend on the dose of the incoming metal. In animals obtaining a combination of the above mentioned metals, a slight potentiation of cumulation was observed. Such an effect can be the consequence of copper and iron interaction during their intestinal absorption. This hypothesis is supported by several articles indicating enhanced iron absorption during copper repletion (Han and Wessling-Resnick, 2002) on the one hand, and by observations of iron deficiency induced by insufficient copper entrance (Reeves, 2004) on the other.

The mechanisms of the observed cumulation of iron in adipose tissue of all experimental animals, including copper-treated animals, require further investigations. Theoretically, such an effect can be the consequence of iron sequestration in adipose-tissue macrophages induced by obesity (Yanoff *et al.*, 2007; Zekanowska *et al.*, 2011).

The observed induction of oxidative stress by iron and copper is a consequence of their prooxidant features, which were shown and discussed in numerous works (Leonard *et al.*, 2004; Jomova and Valko, 2011; Krasikov *et al.*, 2011). Oxidative stress itself exhibits inadequate production of reactive oxygen species and their reactive metabolites (Durackova, 2011) leading to cell damage and consequently to several diseases (Aruoma, 1998; Pryor *et al.*, 2006). Activation of free radical processes stimulates also the development of inflammation in adipose tissue and its growth (Martinez, 2006; Matsuzawa-Nagata *et al.*, 2008). Inflammation is accompanied first by lymphoid and then by macrophagal infiltration in adipose tissue (Kintscher *et al.*, 2008; Nishimura *et al.*, 2009), leading to subsequent intensification of free radical processes (Fantone and Ward, 1982). On the other hand, there is evidence of orexigenic action of copper via stimulation of neuropeptide Y expression in the hypothalamus (Li *et al.*, 2008). These data support our results indicating enhancement of food consumption and weight gain in rats obtaining copper. Potentiation of the above mentioned effects in case of combined consumption of copper and iron is possibly a consequence of two mechanisms acting simultaneously: orexigenic effect of copper and prooxidant effect of iron.

In conclusion, we showed that consumption of drinking water containing iron and copper led to intensified weight gain and increase in adipose tissue mass. Entrance of the metals tested into the organism resulted also in development of oxidative stress in fat pads that can be one of the mechanisms leading to increased adipose tissue mass. Yet elucidation of the delicate mechanisms of the observed effects of copper and iron call for further detailed investigations.
